# Case Report: Atypical Cornelia de Lange Syndrome

**DOI:** 10.12688/f1000research.3-33.v2

**Published:** 2015-05-27

**Authors:** Vito Leanza, Gabriella Rubbino, Gianluca Leanza

**Affiliations:** 1 Surgery Department, Obstetrics and Gynecologic Unit “Ospedale Santo Bambino”, Catania University, Catania, Italy

**Keywords:** facial dysmorphia, ultrasound

## Abstract

Cornelia de Lange Syndrome (CdLS) (also called Bushy Syndrome or Amsterdam dwarfism), is a genetic disorder that can lead to several alterations. This disease affects both physical and neuropsychiatric development. The various abnormalities include facial dysmorphia (arched eyebrows, synophrys, depressed nasal bridge, long philtrum, down-turned angles of the mouth), upper-extremity malformations, hirsutism, cardiac defects, and gastrointestinal alterations. The prevalence of this syndrome is approximately one per 15,000. Ultrasound is not the perfect means to diagnose CdLS, however, many abnormalities can be detected prenatally by scrupulous image observation.

We report an atypical CdLS case characterized by increased nuchal translucency in the first trimester, normal karyotype, saddle nose, micrognathia with receding jaw, low set ears, facies senilis, arthrogryposis of the hands, absence of the Aranzio ductus venous, dilatation of gallbladder and bowel, a unique umbilical artery, increased volume of amniotic fluid, and intrauterine growth retardation ending with the interruption of pregnancy.

## Introduction

Cornelia de Lange Syndrome (CdLS) is a genetic disorder that can lead to several alterations. It affects both physical and neuropsychiatric development. The several abnormalities include facial dysmorphia (arched eyebrows, synophrys, depressed nasal bridge, long philtrum, down-turned angles of the mouth), upper-extremity malformations, hirsutism, cardiac defects, and gastrointestinal alterations ^1^. The prevalence of this syndrome is approximately one per 15,000 ^2^. Many markers have to be considered. Nuchal translucency (NT) measurement during the first trimester screening between 11 and 14 weeks gestation has now been clearly identified as a marker for aneuploidies and in particular for trisomy 21. Even in the absence of aneuploidy, increased foetal nuchal translucency has been shown to be a marker for foetal heart malformations and several other foetal defects linked to genetic syndromes when the measure exceeds the 95 ^th^ percentile (3–5 mm) ^3^. When conventional karyotyping is normal, enlarged NT is a strong marker for adverse pregnancy outcome, associated with miscarriage or intrauterine death). Unspecified genetic syndromes involving developmental delay may only emerge after birth and become evident after the first years of life. Several abnormalities have been reported in foetuses with enlarged NT: the majority are cardiac defects, diaphragmatic hernia, exomphalos, body stalk anomaly, skeletal defects, certain genetic syndromes (such as congenital adrenal hyperplasia), foetal akinesia, deformation sequence, Noonan syndrome, Smith-lemli-Opitz syndrome and spinal muscular atrophy ^4^. The saddle nose, characterized by a markedly depressed bridge has been described in AIDS embryopathy, Christ-Siemans-Touraine syndrome, various deletion syndromes, foetal trimethadione syndrome, Laron-type dwarfism, leprechaunism, multiple epiphyseal dysplasia, otospondylomegaepiphyseal dysplasia (OSMED) syndrome, relapsing polychondritis, thanatophoric dwarfism, Wegener’s granulomatosis, and various conditions that are further characterized by gargoyle-like faces. Micrognathia is a malformation of the foetal face characterized by a small mandible. Micrognathia may be idiopathic but is more commonly associated with many different syndromes. Retrognathia (recession of the chin) is assessed through the measurement of the inferior facial angle, as defined on a mid-sagittal view. With routine ultrasound, the receded chin may be observed on a profile of the face. Yet, this diagnosis is often missed during a routine ultrasound examination. The mandible is known to grow significantly during the third trimester. If mandibular alteration is suspected, particular attention should be paid to the growth of the chin throughout the remainder of the pregnancy. Conditions associated with micrognathia include chromosomal abnormalities, neuromuscular abnormalities, single-gene disorders, and other syndromes. The prognosis of foetal micrognathia is poor, even in chromosomally normal foetuses. Frequent malformations associated with micrognathia are: Pierre Robin sequence (micrognathia, cleft palate, or both) ^5^; Cerebrocostomandibular syndrome (diagnosed on the basis of micrognathia, a posterior cleft palate defect, and characteristic rib gap abnormalities); Cornelia de Lange syndrome, (underdeveloped chin with tetralogy of Fallot, intrauterine growth restriction, and an abnormal right hand) ^6^; hypochondrogenesis type II and Caffey disease.

## Case report

**Figure 1.**
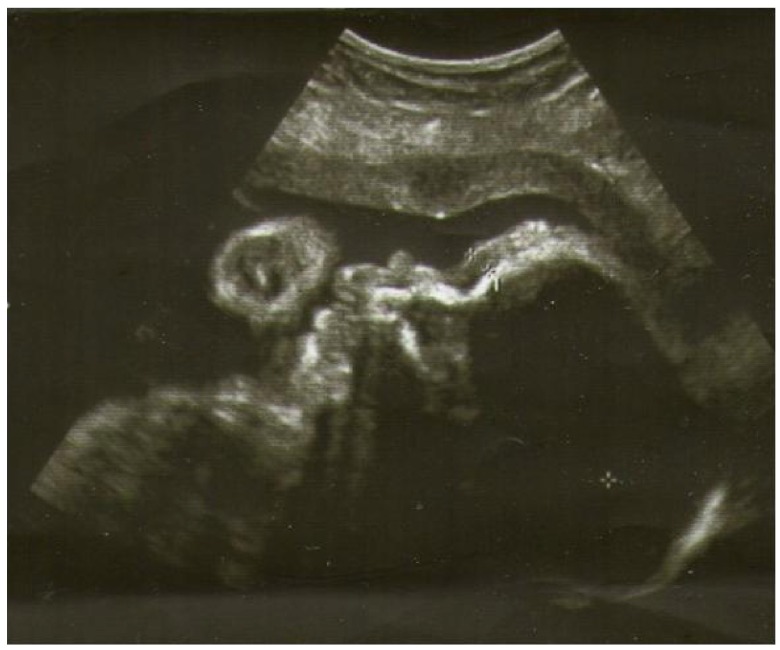
30 ^th^ week face profile with ultrasound.

**Figure 2.**
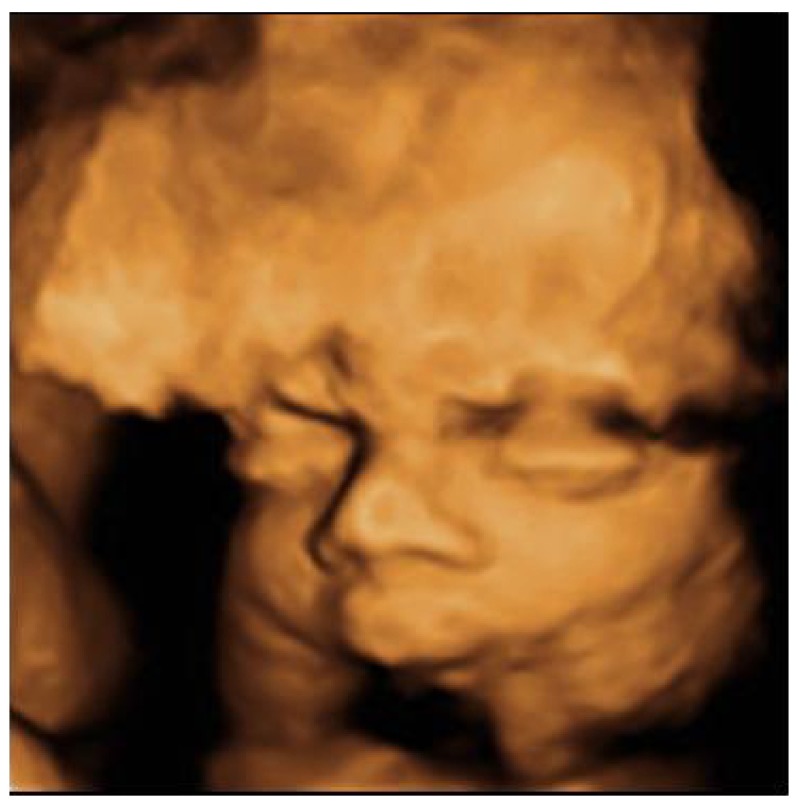
29 ^th^ week face aspect with 3D ultrasound.

We report a case of a healthy 31-year-old, gravida 2, para 1 at 30 weeks of gestation that was admitted to S. Bambino University Hospital in Catania for ultrasound examination. Ultrasounds revealed nuchal oedema, saddle nose, micrognathia with receding jaw, low set ears, facies senilis at 3D ultrasounds, arthrogryposis of the hands, absence of Aranzio’s ductus venous, dilation of gallbladder and bowel, single one umbilical artery, increased volume of amniotic fluid. Intrauterine growth retardation was associated as well. ( Figure 1) Micrognathia was evident on midsagittal and 3D scan. The biparietal diameter was 68 mm, femur length 47 mm, suggesting foetal growth restriction. Pulsed Doppler sonography showed normal middle cerebral artery and umbilical artery pulsatility indices. The obstetric history revealed increased nuchal translucency thickness (NT) at 11 weeks (4 mm, > 95 centile). No genetic alterations were found at the amniocentesis carried out during 16 ^th^ week of pregnancy (normal karyotype of 46, XX). Morphological ultrasound at 22 weeks of pregnancy was not able to detect the syndrome. A further ultrasound examination at 29 weeks ( Figure 2) of pregnancy pointed out a foetal dysmorphism and the pregnant woman asked for our consultation.

**Figure 3.**
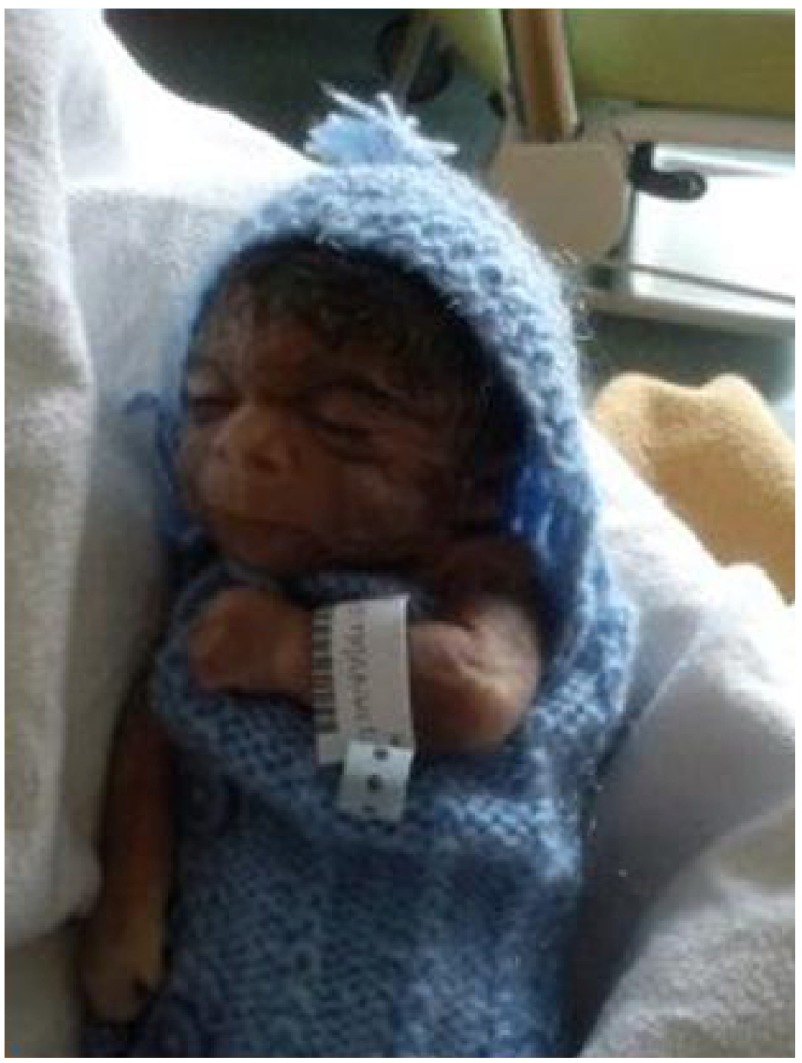
Aspect of the new born (facies senilis).

Soon after, interruption of pregnancy occurred. The autopsy showed a foetus with a weight between the 5 ^th^ and the 10 ^th^ percentile and a dysmorphic syndrome with malformation features amenable to CdLS ( Figure 3). The foetus had a typical dysmorphic face, hirsutism, rhizomelic limb, bilateral camptodactyly, single transverse palmar crease, proximal and very short inches with hypoplasia of 1 ^st^ metacarpal and single phalanx, II-III membranous syndactyly of feet, right diaphragmatic hernia, bilateral cryptorchidism, microcephaly, numerous nodules of heterotopic cerebellar vermis, single umbilical artery and hypotrophic placenta devoid of inflammatory lesions.

## Discussion and conclusion

CdLS is a multisystem malformation syndrome recognized primarily on the basis of the morphological characteristics (malformations of the cranial, cardiac, gastrointestinal, and skeletal systems) ^7^. However, there is wide clinical variability of disorders, with milder phenotypes that may be difficult to ascertain on the basis of physical features. In certain cases the diagnosis may be missed when ultrasound examination is not performed accurately. Criteria to detect CdLS are not standardized. The main alterations are as follows ^9^:

- Facial abnormalities (synophrys, long eyelashes, microcephaly, anteverted nostrils)
- Cardiac defects (defects of ventricles or atria, aortic or pulmonary stenoses, tetralogy of Fallot, atrioventricular canal, single ventricle, aorto-pulmonary window, truncus arteriosus communis)
- Abnormalities of upper limbs (ectrodactylia and monodactylism)
- Gastrointestinal alterations (diaphragmatic hernia)
- Musculoskeletal malformation
- Intrauterine growth retardation

Ultrasound detection of eyelashes is considered a clue for prenatal diagnosis of CdLS, but it can be missed in clinical practice ^10^. CdLS is considered a cohesinopathy. Mutations in cohesin, or its regulators, cause a spectrum of human developmental syndromes. Cohesinopathy disorders include both CdLS and Roberts Syndrome. The discovery of novel roles for chromatid cohesion proteins in regulating gene expression led to the idea that cohesinopathies are caused by dysregulation of multiple genes downstream of mutations in cohesin proteins. Consistent with this idea, there is an altered expression of developmental genes and an incomplete overlap among dysregulated genes in different components of the cohesin apparatus ^11^. CdLS is considered a dominantly inherited congenital malformation disorder, caused by mutations in the cohesin-loading protein *NIPBL* for nearly 60% of individuals. In humans, the multisubunit complex cohesin is made up of *SMC1, SMC3, RAD21* and a *STAG* protein. These form a ring structure that is proposed to encircle sister chromatids to mediate sister chromatid cohesin; it also has a key role in genetic regulation. In CdLS cell lines an altered transcription with either *NIPBL* or *HDAC8* mutations has been found ^12^. The proteins produced from mutated genes interfere in the foetal development. Within cells, these proteins help regulate the structure and organization of chromosomes and are involved in the repair of damaged DNA. They also regulate the activity of certain genes in the developing limbs, face, and other parts of the body. Researchers are looking for additional changes in the *NIPBL*, *SMC1A* and *SMC3* genes, as well as mutations in other genes, that may be responsible for this condition. The majority of cases result from new gene mutations and occur in people with no family history. CdLS can be prenatally diagnosed in a family with a known mutation in a CdLS gene. The characteristic ultrasonographic profile may allow for prenatal diagnosis of CdLS in subsequent pregnancies for couples with a prior child with CdLS in whom a mutation has not been identified or when there are unexplained signs of foetal abnormality during pregnancy, such as oligo- or polyhydramnios, a low maternal serum PAPP-A level and/or increased NT, foetal growth retardation, or structural anomalies consistent with CdLS ^13^. Data from mutational testing on known CdLS genes ( *NIPBL, SMC1A, SMC3, RAD21*, and *HDAC8*) are important in the diagnosis of the typical syndrome. Indeed, the article published by Clark *et al.* (2012) ^13^ recommends molecular analysis of CdLS genes to prenatal diagnosis of CdLS. In our case data from mutational testing on known CdLS genes ( *NIPBL, SMC1A, SMC3, RAD21*, and *HDAC8*) was negative and so we consider this a case of atypical CdLS. Mutations in *NIPBL* are not present in all cases and they account for about 60% of CdLS; while mutations in *SMC1A* and *SMC3* only for a small percentage ^14^. In some cases the diagnosis is made prenatally in other cases the syndrome is diagnosed after childbirth. Currently, there are no definitive prenatal screening measures that lead to the diagnosis of CdLS ^13^. Fewer than 1% of individuals diagnosed with CdLS have an affected parent ^14^. Recommendations for the evaluation of parents of a proband with an apparent *de novo* mutation include clinical examination for features of CdLS as well as the plotting of the growth parameters and the molecular genetic testing for *NIPBL* mutation compared to the one identified in the proband. In our atypical Cornelia de Lange syndrome case molecular tests, conducted on the blood samples of the parents in a sequential manner of for NIPBL, SMC1A, SMC3, RAD21 and HDAC8, were negative. The diagnosis is indicated when there is a major longitudinal deficiency defect of the upper limb, severe prenatal and postnatal growth retardation, and severe mental retardation. Features used to make the correct diagnosis include penciled and arched eyebrows, a high set/short anteverted nose, a long flat philtrum, a thin upper lip, downturned corners of the mouth, and micrognathia. Characteristics that are misleading include full or flat brows, a prominent nasal bridge or bulbous tip, and/or a normal or prominent chin. The genetic tests with positive results confirm the presence of the syndrome while the negative results do not exclude it. Indeed, mutations in *NIPBL, SMC1A* and *SMC3* are not present in all cases of CdLS as in our case. Less than 1% of individuals diagnosed with CdLS have an affected parent ^14^. As genetic and biochemical tests are unreliable at present, antenatal detection depends upon identification of some aspects of the phenotype in the foetus using ultrasound imaging ^15, 16^. When the syndrome is not recognized during pregnancy, the newborn may survive with a low quality of life and, thus, medical team could become involved in surgical procedures ^17^. Each malformation causes an impact in the psychological sphere of both the individual and the family ^18^. Last but not the least, the failure of an early diagnosis may lead to medical-legal issues ^19– 21^.

## Consent

Written informed consent for publication of clinical details and clinical images was obtained from the patient and her husband.
