# Supplementary material for: Case Report: Atypical Cornelia de Lange Syndrome
Source: F1000Res. 2015 May 27;3:33. Originally published 2014 Jan 31. [Version 2] doi: 10.12688/f1000research.3-33.v2 (PMC4706058; doi:10.12688/f1000research.3-33.v2)
Supplement: Peer review history

# Peer review: Referee response for version 2

**DOI:** 10.5256/f1000research.4647.r4026

**Published:** 2015-12-10

**Authors:** Orkun Çetin

**Affiliations:** 1 Obstetrics and Gynecology, Istanbul Suleymaniye Birth and Gynecology Training and Research Hospital, Istanbul, Turkey

I appreciate your work and thank you for your effort. However I have some considerations to be clarified.

1. The manuscript needs a light sub-edit for the English.
2. The authors made the final diagnosis only with the clinical findings. However, there were not any photos that confirmed CdLS. If possible, the autopsy photos should be added to the manuscript.

The manuscript could be accepted after minor revisions.

I have read this submission. I believe that I have an appropriate level of expertise to confirm that it is of an acceptable scientific standard, however I have significant reservations, as outlined above.

# Peer review: Referee response for version 2

**DOI:** 10.5256/f1000research.4647.r8779

**Published:** 2015-05-28

**Authors:** Laird Jackson

**Affiliations:** 1 Department of Obstetrics and Gynecology, Drexel University, Philadelphia, PA, USA

The authors have responded appropriately to the earlier comments except in one instance.  It may be due to language differences but the term "unreliable" should not be used to describe the genetic tests referred to in the paper.  The tests are "reliable" if performed properly; in that they do or do not demonstrate a copy-number change (if by microarray) or a point mutation (if by some means of genome sequencing.  What the authors intend to say is that a negative test (i.e. one that does not demonstrate a molecular change), is not equivalent to ruling out the clinical diagnosis.  So they might simply say that the molecular tests are only "conclusive" in about 60-70% of the clinically recognized cases.

I have read this submission. I believe that I have an appropriate level of expertise to confirm that it is of an acceptable scientific standard.

# Peer review: Referee response for version 1

**DOI:** 10.5256/f1000research.1212.r4483

**Published:** 2014-04-14

**Authors:** Laird Jackson

**Affiliations:** 1 Department of Obstetrics and Gynecology, Drexel University, Philadelphia, PA, USA

I agree with the report and comments of Dr Jinglian Liu, who has also reviewed this article. The case report is interesting in its detail of the prenatal ultrasound examination and observations, but completely fails its responsibility to connect those observations to appropriate use of contemporary molecular diagnostic opportunities. As such, this article fails the medical community by not accepting the opportunity to connect their useful prenatal morphological observations to detectable objective molecular findings.

I have read this submission. I believe that I have an appropriate level of expertise to confirm that it is of an acceptable scientific standard, however I have significant reservations, as outlined above.

# Peer review: Referee response for version 1

**DOI:** 10.5256/f1000research.1212.r3450

**Published:** 2014-02-14

**Authors:** Jinglan Liu

**Affiliations:** 1 Department of Pediatrics, Drexel University, Philadelphia, PA, USA

This manuscript describes a fetus with clinical features suggestive of Cornelia de Lange Syndrome (CdLS).

My comments are as follows:

1. Data from mutational testing on known CdLS genes ( *NIPBL, SMC1A, SMC3, RAD21*, and *HDAC8*) would be important for this case.  The article published by Clark *et al.* (2012) which was also cited by the authors, recommends molecular analysis of CdLS genes to prenatal diagnosis of CdLS.
2. The authors should compare prenatal findings in the current case with what described in the article by Clark *et al. (*2012). Without molecular testing data, I would like to see a table demonstrating the current case presenting prenatal clinical features consistent with what has been documented in the literature by studying  a larger cohort of probands.  
3. In the discussion, in the beginning of first paragraph, the authors claim that CdLS is diagnosed primarily by morphological characteristic and cited a paper published in 1933. At present, the diagnosis criteria of CdLS cannot be the same as what was 90 years ago, particularly with the identification of specific gene mutations in CdLS probands.  Please re-phrase this part, and cite updated references.
4. In the end of the discussion, what does it mean “genetic tests are not reliable”?  As a matter of fact, molecular testing has become a widely accepted concept and a popular diagnostic approach for individuals with congenital anormalies.    

Minor things:

1. OMIM numbers of CdLS and disease genes should be given.
2.  Please double check the spelling and eliminate typographic errors, also, some terms are not accurately used.  Below is listed some examples of those errors:

- Abstract – “kariotype” -> “karyotype”
- Introduction – “psychical” - > I’d like to use “neuropsychiatric”
- 2 ^nd^ paragraph in case report – “dimorphism” -> “dysmorphism”
- 2 ^nd^ paragraph in discussion – “cohesion”  -> “cohesin”
- 2 ^nd^ paragraph in discussion – please italicize the gene names  “NIPBL” and “HDAC8”
- 2 ^nd^ paragraph in discussion – “familiarity” ? What does it mean?  Why not change to “ no family history”

I have read this submission. I believe that I have an appropriate level of expertise to confirm that it is of an acceptable scientific standard, however I have significant reservations, as outlined above.

# Response

**Published:** 2014-09-01

**Authors:** vito leanza

**Affiliations:** Italy

1. *“Data from mutational testing on known CdLS genes (NIPBL, SMC1A, SMC3, RAD21, and HDAC8) would be important for this case. The article published by* *Clark et al. (2012)* *which was also cited by the authors, recommends molecular analysis of CdLS genes to prenatal diagnosis of CdLS.”*
  In our case report, data from mutational testing on known CdLS genes ( *NIPBL, SMC1A, SMC3, RAD21*, and *HDAC8*) are negative , indeed it was called atypical Cornelia de Lange Syndrome (CdLS).
  Mutations in *NIPBL* are not present in all cases and they account for about 60% of CdLS; mutations in *SMC1A* and *SMC3* account for a small percentage (Deardorff *et al.*, 2011).
2. *“The authors should compare prenatal findings in the current case with what described in the article by* *Clark et al. (2012)* *.* *Without molecular testing data, I would like to see a table demonstrating the current case presenting prenatal clinical features consistent with what has been documented in the literature by studying a larger cohort of probands.”*
  Cornelia de Lange Syndrome (CdLS) is a multisystem developmental disorder characterized by growth retardation, cognitive impairment, external and internal structural malformations, and characteristic facial features. Currently, there are no definitive prenatal screening measures that lead to the diagnosis of CdLS. ( Clark *et al. *2012).
  In our case (atypical CdLS)  increased nuchal translucency in the first trimester, normal karyotype, saddle nose, micrognathia with receding jaw, low set ears, facies senilis, arthrogryposis of the hands, absence of the Aranzio ductus venous, dilatation of gallbladder and bowel, a unique umbilical artery, increased volume of amniotic fluid, and intrauterine growth retardation were found.
  Fewer than 1% of individuals diagnosed with Cornelia de Lange syndrome have an affected parent.
  Recommendations for the evaluation of parents of a proband with an apparent *de novo* mutation include clinical examination for features of CdLS, complete with plotting of growth parameters and molecular genetic testing if the NIPBL mutation has been identified in the proband. In our atypical Cornelia de Lange syndrome molecular tests of parents were negative.
3.  
*“In the discussion, in the beginning of first paragraph, the authors claim that CdLS is diagnosed primarily by morphological characteristic and cited a paper published in 1933. At present, the diagnosis criteria of CdLS cannot be the same as what was 90 years ago, particularly with the identification of specific gene mutations in CdLS probands. Please re-phrase this part, and cite updated references.”*
  Diagnosis is based on clinical findings. High-resolution ultrasound examination to follow growth and to evaluate the limbs, heart, diaphragm, palate, and other organs or structures affected in CdLS is fundamental. Reported prenatal ultrasound findings include: increased nuchal translucency in the first trimester [ Sekimoto *et al.*, 2000; Huang & Porto, 2002]; Growth failure, which typically presents in the second trimester; and the typical *in utero* facial profile of a fetus with CdLS consisting of micrognathia, a prominent upper lip, and a depressed nasal bridge with somewhat anteverted nares [ Ranzini *et al*., 1997; Boog *et al.,* 1999; Urban & Hartung, 2001].
4. *“In the end of the discussion, what does it mean “genetic tests are not reliable”? As a matter of fact, molecular testing has become a widely accepted concept and a popular diagnostic approach for individuals with congenital anormalies.”*
  Genetic tests are not reliable when negative. On the contrary when positive they are important for syndrome.
  Indeed, mutations in NIPBL  are not present in all cases and they account for about 60% of CdLS; mutations in SMC1A and SMC3 account for a small percentage ( Deardorff *et al.*, 2011).

Minor things:

These minor things are easy to correct. I thank the first referee for his clarifications.
